# Dendritic Cell Cancer Therapy: Vaccinating the Right Patient at the Right Time

**DOI:** 10.3389/fimmu.2018.02265

**Published:** 2018-10-01

**Authors:** Wouter W. van Willigen, Martine Bloemendal, Winald R. Gerritsen, Gerty Schreibelt, I. Jolanda M. de Vries, Kalijn F. Bol

**Affiliations:** ^1^Department of Medical Oncology, Radboud University Medical Center, Nijmegen, Netherlands; ^2^Department of Tumor Immunology, Radboud Institute for Molecular Life Sciences, Nijmegen, Netherlands

**Keywords:** dendritic cell, vaccination, immunotherapy, checkpoint inhibitor, cancer, adjuvant

## Abstract

Immune checkpoint inhibitors propelled the field of oncology with clinical responses in many different tumor types. Superior overall survival over chemotherapy has been reported in various metastatic cancers. Furthermore, prolonged disease-free and overall survival have been reported in the adjuvant treatment of stage III melanoma. Unfortunately, a substantial portion of patients do not obtain a durable response. Therefore, additional strategies for the treatment of cancer are still warranted. One of the numerous options is dendritic cell vaccination, which employs the central role of dendritic cells in activating the innate and adaptive immune system. Over the years, dendritic cell vaccination was shown to be able to induce an immunologic response, to increase the number of tumor infiltrating lymphocytes and to provide overall survival benefit for at least a selection of patients in phase II studies. However, with the success of immune checkpoint inhibition in several malignancies and considering the plethora of other treatment modalities being developed, it is of utmost importance to delineate the position of dendritic cell therapy in the treatment landscape of cancer. In this review, we address some key questions regarding the integration of dendritic cell vaccination in future cancer treatment paradigms.

## Introduction

Since William Coley made his early contributions to the study of cancer immunotherapy in the 1890s, harnessing the capabilities of the immune system to eliminate cancer cells remained a long-sought dream (1). In the last decade, efforts to realize this dream were finally rewarded with the introduction of immune checkpoint inhibitors (ICI). ICI showed the feasibility of immunotherapy and revolutionized the treatment of cancer. The success of ICI spurred a considerable amount of research activity into the field of immunotherapy. Despite its resounding success, ICI still have two important limitations: they are associated with significant (immune-related) toxicity and a portion of patients does not respond (2–7). Immunotherapy however, encompasses more than ICI alone. Dendritic cell (DC) vaccination is an alternative form of immunotherapy and is a prime candidate to enrich the treatment possibilities for cancer. Considering the fact that the field of immunotherapy is a fast-moving field, it is of utmost importance to delineate the position of DC vaccines in the therapeutic landscape of cancer. In this review, we will explore some important questions regarding this position, with the focus on four malignancies (glioblastoma, melanoma, prostate cancer, and renal cell carcinoma) in which phase III trials with DC vaccines have been performed or are ongoing.

### The evolving field of immune checkpoint inhibition

Currently, the clinical application of immunotherapy is mainly defined by ICI. ICI target immune checkpoint molecules such as CTLA-4, PD-L1, and PD-1. These molecules have immune response inhibiting functions and are involved in the prevention of autoimmunity and the maintenance of peripheral tolerance. It is well known that tumor cells are able to upregulate the expression of checkpoint molecules, leading to anergy of cytotoxic T-cells in the tumor microenvironment. CTLA-4, PD-L1, and PD-1 have distinct functions; CTLA-4 exerts its inhibitory functions on the initial T-cell activation whereas PD-1 and PD-L1 have roles in the inhibition of the effector functions of T-cells (8, 9). ICI antagonize these molecules and thereby aim to augment the anti-cancer immune response.

In 2010, ipilimumab (a monoclonal antibody targeting CTLA-4) was the first immunotherapeutic agent providing clinical benefit in cancer patients, extending median overall survival (OS) to 10 months (compared to 6.4 months for the control group receiving a gp100 peptide vaccine) in metastatic melanoma (3). With an overall response rate (ORR) of ~10–20%, ipilimumab was a great improvement compared to the standard of care at the time, but it still offers clinical benefit in only a portion of melanoma patients (10, 11). However, in a substantial portion of responding patients, clinical benefit is durable (5). In 2014, two monoclonal antibodies (pembrolizumab and nivolumab) targeting the PD-1 pathway were also approved for the treatment of metastatic melanoma. Compared to ipilimumab, anti-PD-1 inhibition achieves a higher ORR of ~40% (4, 5, 12, 13).

After these landmark studies, research into ICI accelerated. With the addition of PD-L1 targeting agents avelumab, atezolimumab, and durvalumab, the field of ICI now encompasses six FDA and EMA-approved monoclonal antibodies (mAb) (14, 15, 16). Most of these ICI are approved for the treatment of multiple malignancies (Table 1). The number of approved indications of these mAb is likely to grow as they are currently tested in a large number of additional malignancies (17).

**Table 1:** Indications of the six currently approved monoclonal antibodies in the treatment of cancer (as of May 2018).

**Monoclonal antibody**	**Target**	**FDA/EMA-approved indications**
Ipilimumab	CTLA-4	Melanoma
Nivolumab	PD-1	Melanoma, NSCLC, RCC, urothelial carcinoma, MSI-high/dMMR CRC, HCC, Hodgkin's lymphoma, HNSCC
Pembrolizumab	PD-1	Melanoma, NSCLC, HNSCC, urothelial carcinoma, Hodgkin's lymphoma, MSI-high cancer, gastric/gastroesophageal cancer
Avelumab	PD-L1	Merkel cell carcinoma, urothelial carcinoma
Atezolimumab	PD-L1	Urothelial carcinoma, NSCLC
Durvalumab	PD-L1	Urothelial carcinoma, NSCLC
Combined treatment with ipilimumab and nivolumab	CTLA-4/PD-1	Melanoma, RCC

*CRC, colorectal cancer; CTLA-4, cytotoxic T-lymphocyte-associated protein 4; HCC, hepatocellular carcinoma; HNSCC, head and neck squamous cell carcinoma; dMMR, DNA mismatch repair deficiency; MSI, microsatellite instability; NSCLC, non-small-cell lung carcinoma; PD-1, programmed cell death protein; PD-L1, programmed death-ligand 1; RCC, renal cell carcinoma*.

Besides PD-1, PD-L1 and CTLA-4, other checkpoint molecules (such as TIM-3 and LAG-3) have shown to inhibit the anti-cancer immune response (18). Several mAb targeting these alternative checkpoint molecules are in various stages of clinical investigation. Therefore, it is expected that the number of clinically available mAb will be further expanded (17). In addition to the treatment of metastatic disease, research is moving toward the application of ICI in the adjuvant treatment of cancer. For example, adjuvant ipilimumab, nivolumab, and pembrolizumab after surgically resected stage III melanoma recently have shown to improve progression-free survival (PFS) and in case of adjuvant ipilimumab, an prolonged OS was seen (19–21).

ICI come with a different toxicity profile compared to other anti-cancer therapeutics, caused by specific immune-related side effects. Monotherapy with anti-PD-1 mAb and anti-CTLA-4 mAb are associated with 10–16% and 30–40% grade 3 or 4 adverse events, respectively (3, 5, 6, 11, 22). In contrast, DC vaccination is associated with little toxicity as grade 3 or 4 adverse events are very uncommon (23–25). In addition, the application of DC vaccination might further improve response rates on ICI.

### Dendritic cell vaccination

Since their discovery by Steinman in 1973, it became clear that DC are antigen-presenting cells crucial in activating the adaptive immune system (26). DC are spread throughout the body, constantly monitoring their surroundings for antigens and danger signals. Once stimulated by an activating stimulus, they undergo maturation and migrate to lymphoid organs where they activate several effector cells of the immune system, primarily T-cells and B-cells (27).

**Figure 1:**
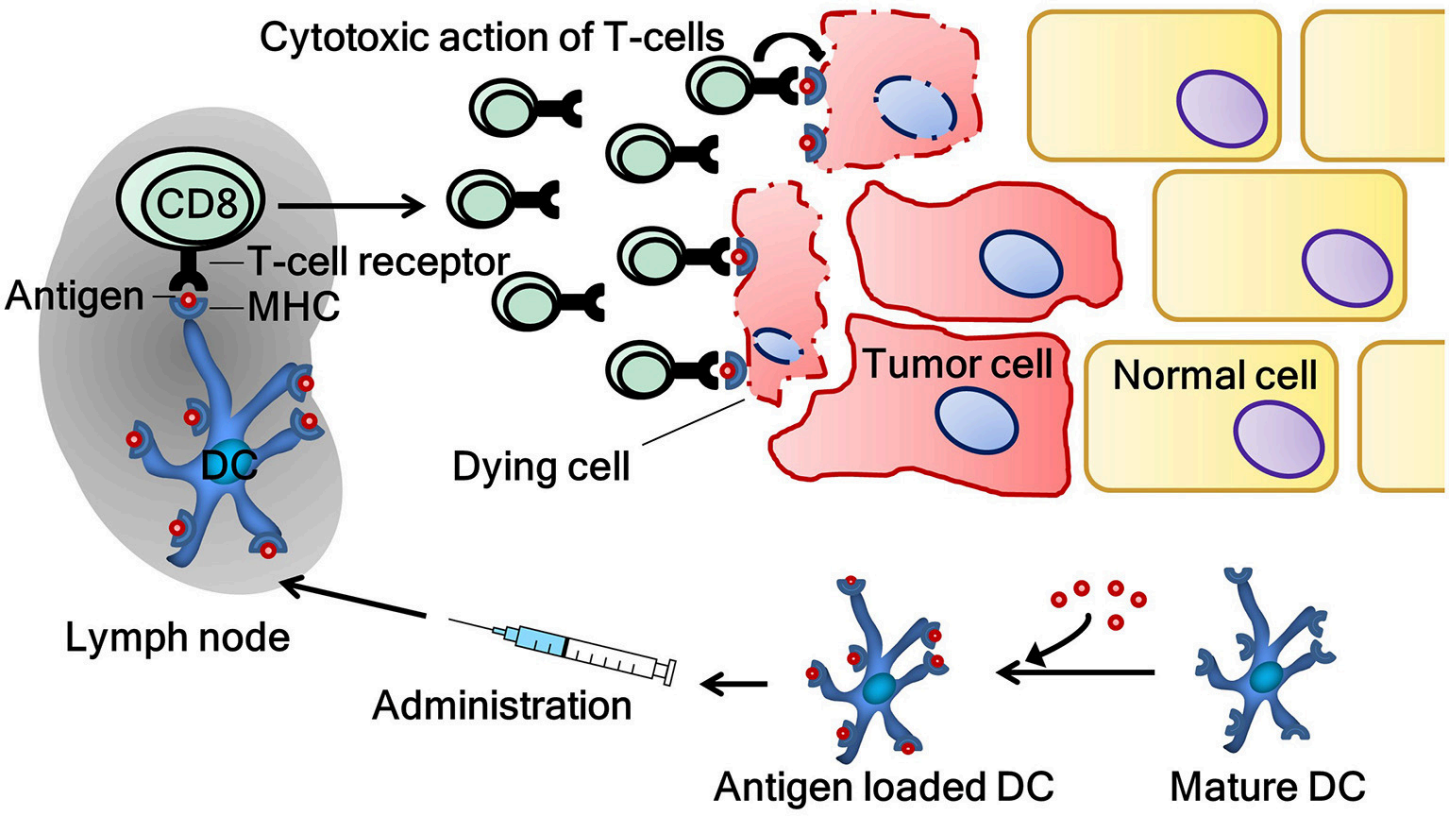
The induction of a tumor-specific immune response by dendritic cell vaccination. Tumor antigen-specific T-cells are activated by dendritic cells, which are *ex vivo* loaded with tumor antigen(s). Activated T-cells subsequently patrol the body in search of their respective antigen. When their target is found, T-cells exert their cytotoxic functions on cancer cells. CD8, cluster of differentiation 8 (cytotoxic T-cell); DC, dendritic cell; MHC, major histocompatibility complex.

Through this process, DC are vital for immunosurveillance. Immunosurveillance signifies the crucial role of the immune system in the detection and elimination of both pathogens and cancer cells. However, the development of malignancy is an indolent process in its early stages, therefore, immunosurveillance occasionally fails. At an early stage, tumors sometimes silence an initiated immune response or fail to express the “danger signals” necessary for the activation of the immune system. When the process of immunosurveillance fails, one of the hurdles for the outgrowth of cancer cells is omitted. DC vaccination aims to correct this failure by reversing the ignorance of the immune system to malignant cells. To achieve this, DC are stimulated *ex vivo* with danger signals and loaded with tumor-specific antigen(s) on their major histocompatibility complex molecules with the intent of activating antigen-specific T-cells which selectively eliminate antigen-bearing cancer cells (Figure 1). The majority of research groups, including our own, employ treatment schemes with multiple administrations of DC vaccine to induce immunological memory (28).

**Figure 2:**
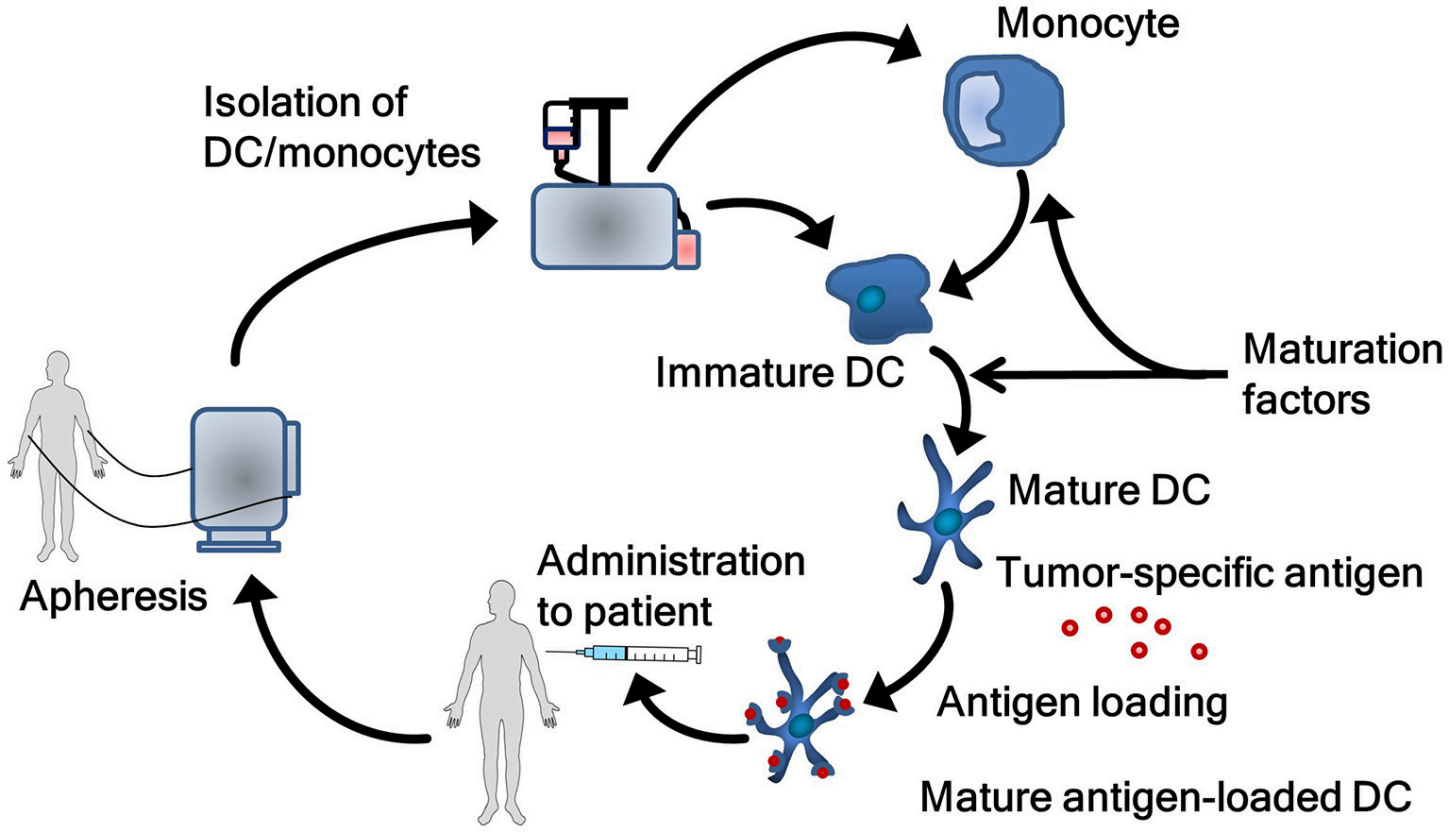
The process of generating dendritic cell vaccines. Autologous dendritic cells or monocytes are obtained via an apheresis procedure. Monocytes first have to be differentiated into dendritic cells. Subsequently, dendritic cells are matured and loaded with tumor antigen. Finally, the dendritic cells are administrated to the patient. DC, dendritic cell.

DC vaccines are produced following some basic principles (Figure 2). Natural circulating DC or monocytes are isolated from autologous peripheral blood mononuclear cells obtained by apheresis. In case of monocytes, *ex vivo* differentiation into DC are required. Both natural circulating DC and monocyte-derived DC are matured as this is essential for effective T-cell activation. Maturation is associated with functional and morphological changes in DC. Following maturation, DC show enhanced expression of major histocompatibility complexes I and II, co-stimulatory molecules and increased capability of cytokine production. These processes are vital, as not or incompletely matured DC can induce tolerance rather than immunity (29). During the process of vaccine manufacturing, DC are loaded with relevant tumor antigen(s) to induce a tumor-specific immune response in the patient. As with the other steps in the process of manufacturing DC, several methods to load DC with antigen exist (30). After quality control, vaccines are administered to the patient.

Despite these basic principles, protocols describing the specific details of DC vaccination manufacturing in trails vary widely. Differences in these protocols cover all aspects of DC vaccination including culture methods, the usage of DC subsets, maturation methods, antigen loading techniques, used antigens and the route of administration. Especially, the subset of DC used, the method of maturation and the choice of antigen(s) are subject of intense research. For example, several groups, including our own, use natural circulating DC instead of monocyte-derived DC. Natural circulating DC do not require extensive culturing which is believed to retain their functionality. Different maturation techniques are also being explored, such as the use of toll-like receptor ligands or electroporation with mRNA-encoding proteins that induce DC maturation (31, 32). Another exciting recent development is the use of neoantigens, which are newly, formed antigens generated from tumor-specific mutated genes, for loading on DC (33). Finally, a more recent development is the recognition that DC, in addition to immune-activating properties, can acquire effector functions (so called killer-DC) following triggering with several differentiating and maturating agents such as interferon (IFN) or lipopolysaccharide (34). Despite these developments, addressing the differences in the generation and production of DC vaccines extensively is beyond the scope of this review.

Regardless of the precise protocol employed, DC vaccination is associated with a very favorable toxicity profile. The majority of side effects reported in various clinical trials were short-lived grade 1 or 2 adverse events, consisting of self-limiting flu like symptoms, fever and local injection site reactions. Treatment-related grade 3 or 4 adverse events following DC vaccination as standalone therapy are uncommon (23, 24).

The goal of DC vaccination is to kill tumor cells by the generation of functional antigen-specific T-cells (23). Despite the challenges associated with measuring the immunological effect of DC vaccination, immunological endpoints are reported in a substantial portion of phase I/II clinical DC vaccination trials using various methods. Several studies even report the generation of antigen-specific T-cells to be positively correlated with survival, strengthening the believe that DC vaccination can result in clinical benefit (25, 35, 36).

Besides the generation of T-cells, intense research is ongoing to find biomarkers, not only for DC vaccination but for immunotherapy in general. Considering ICI treatment, research into predictive biomarkers has revealed several biomarkers predictive for response on ICI (such as mutational burden, PD-L1 expression, and others) (37, 38). Similarly, an example of a predictive biomarker prior to the start of therapy correlated with clinical outcome after DC vaccination is the immune landscape of tumors (39). Up until now, however, biomarkers cannot reliably guide treatment decisions in the clinic for neither ICI nor other forms of immunotherapy, probably owing to the fact that a functional immune response is a complex and multi-step process (40).

### The role of ICI and DC vaccination in metastatic disease

Response rates to DC vaccination vary among cancer types with most studies showing response rates between 10 and 15% (24). Most clinical studies concerning DC vaccination were performed in patients with metastatic disease. Although head-to-head comparisons are not available, ICI achieve superior clinical benefit compared to DC vaccination in most malignancies. In particular for metastatic melanoma and metastatic renal cell carcinoma (RCC), ICI compare favorably in terms of response rates (approximate ORR on anti-PD-1 mAb in RCC: 25%; in melanoma: 40 and 58% when combined with anti-CTLA-4 mAb) (4, 10, 11, 41). ORR in RCC and melanoma patients after treatment with DC vaccines is less, 12 and 9%, respectively (24). Even more important, whereas overall survival benefit for patients with metastatic RCC and metastatic melanoma after ICI treatment is well established, the OS gain for these patients after DC vaccination is less clear (3, 11, 24, 41).

The immunotherapeutic landscape of metastatic castration-resistant prostate cancer (mCRPC) is very different from that of metastatic RCC and metastatic melanoma. Two phase III trials investigating ipilimumab showed, both in pre-docetaxel and post-docetaxel setting, no improvement in OS compared to their control groups (42, 43). Pembrolizumab has shown clinical activity in patients with any type of cancer bearing DNA mismatch repair deficiency (dMMR) and/or microsatellite instability. Individual reports of clinical benefit on anti-PD-1 mAb for patients with dMMR prostate cancer do exist. Unfortunately, dMMR is present in only about 5% of mCRPC patients (44–47). Similar to patients with dMMR, ICI possibly provide benefit in other subgroups of mCRPC patients. For example, nivolumab combined with ipilimumab was tested on patients with an ARV7 mutation which predisposes for a more aggressive form of prostate cancer. In this study, 4 out of 15 patients showed clinical benefit (47). In addition, pembrolizumab has shown some efficacy in a group of patients who progressed after enzalutamide treatment. In a trial of 20 patients, 11 had a partial response or stable disease (45). These patients might be more susceptible to PD-1 antibodies, as PD-1 was shown to be upregulated on DC in patients progressing after enzalutamide (46). After the failure of ipilimumab in prostate cancer patients, a delay in designing new studies with ICI occurred. Currently, ~35 clinical studies with ICI are enlisted for prostate cancer, usually as combination therapies.

Notably, sipuleucel-T gained approval for the treatment of asymptomatic or minimal symptomatic mCRPC. Sipuleucel-T is manufactured from autologous mononuclear cells obtained via apheresis. These cells are incubated with PA2024, a fusion protein of the tumor antigen prostatic acid phosphatase (PAP) and granulocyte-macrophage colony-stimulating factor (GM-CSF). As DC are not specifically isolated from the apheresis product and the end product contains a variety of cells, sipuleucel-T should strictly speaking not be regarded as a pure DC vaccine. Despite this, sipuleucel-T is generally addressed as a DC based-vaccine and is considered to be the first DC-based therapy approved by the FDA. The approval of sipuleucel-T followed the results of a phase III trial including 512 mCRPC patients. The median survival was prolonged with 4 months compared to placebo (48). Another smaller phase III study confirmed these favorable results (49).

Initial enthusiasm about sipuleucel-T has somewhat subsided in recent years since labor intensive production resulted in a highly priced cellular product (around $125.000). At the moment, sipuleucel-T is only available in the USA as market authorization was not granted by the EMA. Recently, a Chinese conglomerate (Sanpower) acquired Dendreon (producer of sipuleucel-T) for over $800 million with the intention to extend the market to Asia. Sipuleucel-T enhanced immune responses toward its antigen (PAP/PA2024). A PAP/PA2024-specific immune response (which is defined as the generation of antigen-specific antibodies, antigen-specific T-cell activation and/or antigen-specific T-cell proliferation) was seen in 79% of patients. The immune responses correlated with OS and could be beneficial for the response on subsequent or concomitant immunotherapeutics, a paradigm which will be detailed in the final chapter of this review (50).

In conclusion, in metastatic malignancies such as non-small-cell lung cancer, melanoma, urothelial cancer and RCC, where ICI are particularly effective, it is unlikely DC vaccination will gain a role as monotherapy in widespread metastatic disease due to its less established clinical benefit.

### Rationale for DC vaccination in the adjuvant treatment of cancer

**Figure 3:**
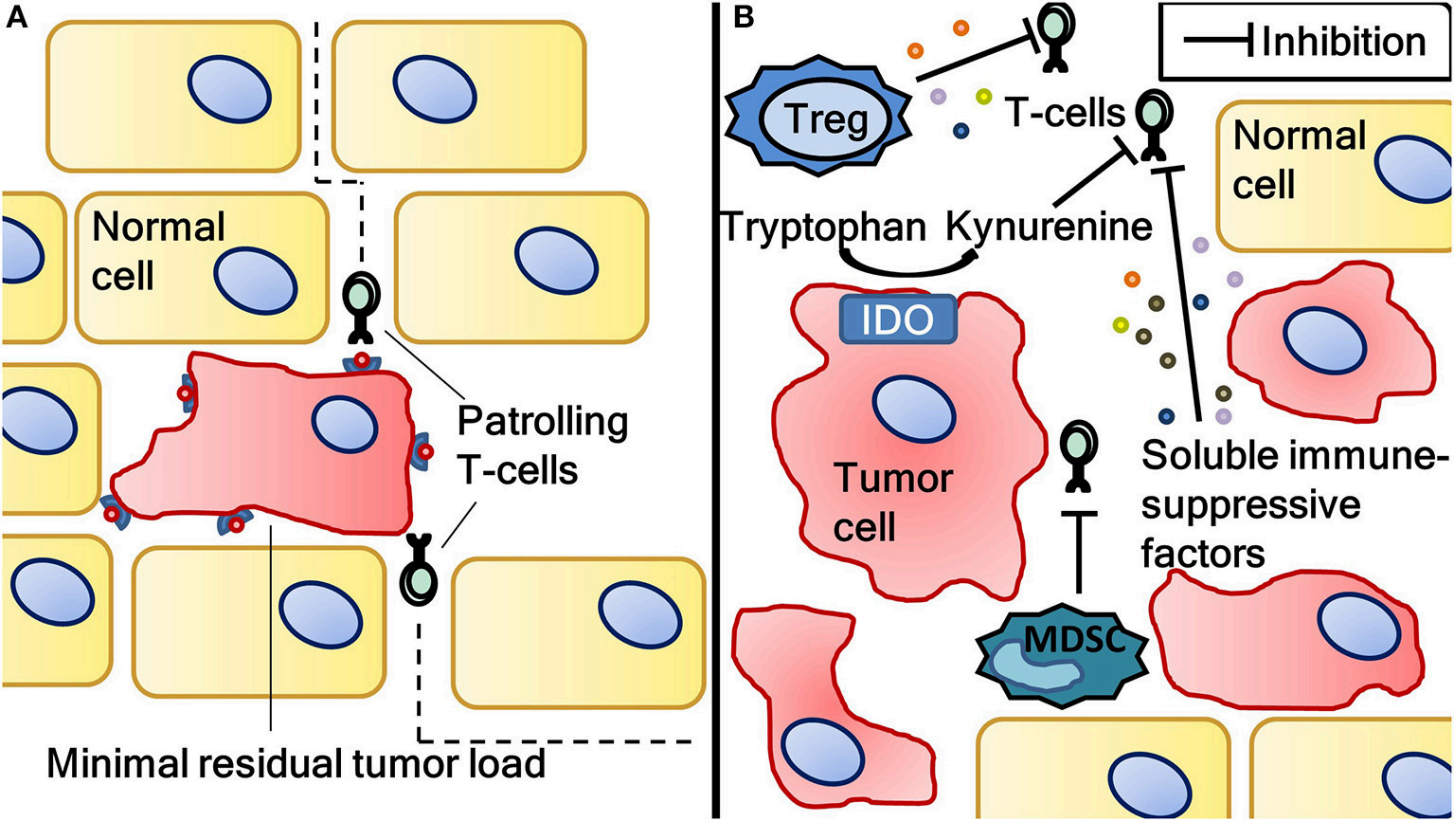
The difference in tumor load-associated immune suppression between minimal residual disease and a situation with high tumor load. Antigen-specific T-cells induced by dendritic cell vaccination eliminating minimal residual disease after surgical resection of cancer **(A)**. Minimal residual disease is associated with less immune suppression as opposed to a situation with more tumor load **(B)**. Tumor load-associated immune suppression is caused by (among other factors) regulatory T-cells, myeloid derived suppressor cells, soluble immune suppressive factors (such as IL-10, TGFβ and VEGF) and indoleamine 2,3-dioxygenase activity. Vaccination-induced T-cells can be rendered anergic by this immune suppression, resulting in inferior clinical results. Therefore, dendritic cell vaccination might be more effective in the adjuvant setting. IDO, indoleamine 2,3-dioxygenase; MDSC, myeloid derived suppressor cells; Treg, regulatory T-cells.

Besides the application of anti-cancer therapeutics in the treatment of metastatic disease, the adjuvant treatment of patients after surgery of local disease is also common practice in oncology. Surgical resection with curative intent aims to excise all tumor burden. However, depending on the type of malignancy, occult residual disease remains in a variable portion of patients and can eventually lead to relapse (51). Adjuvant treatment aims to kill cancer cells, thereby reducing the chance of relapse. With advancing knowledge of the interaction between the immune system and cancer, it becomes increasingly clear that higher tumor load is associated with higher tumor-induced immune suppression. For example, regulatory T-cells (Treg) and myeloid derived suppressor cells (MDSC) attracted by tumor cells induce anergy in T-cells (52). Moreover, several soluble factors secreted by tumor cells, such as TGF-β, IL-10 and VEGF, are recognized to suppress infiltrated effector T-cells (53–55). Also, tumors are able to upregulate indoleamine 2,3-dioxygenase (IDO) which converts tryptophan to kynurenine, inhibiting effector T-cells through a mechanism not completely understood (56). Tumor load-associated immune suppression is generally regarded as the underlying cause of the low clinical response to DC vaccination in metastatic disease (57). Indeed, in our group we detected antigen-specific T-cells in 71% of melanoma patients following adjuvant DC vaccination compared to 23% following vaccination in the metastatic setting (58, 59). In the adjuvant setting, the possibly remaining occult disease represents a low tumor burden, and hence less immune suppression (Figure 3). Therefore, DC vaccination may be more successful in the adjuvant compared to the metastatic setting.

There are some additional arguments to consider DC vaccination as an adjuvant treatment option. Besides efficacy, a low toxicity profile is an important hallmark of any adjuvant treatment as a substantial portion of cancer patients receiving adjuvant treatment would not endure a relapse even without this adjuvant therapy. As noted before, DC vaccination is associated with little toxicity, not only compared to chemotherapy but also compared to ICI. In addition, besides a direct clinical benefit for patients, adjuvant DC vaccination might also prove to be beneficial in improving response to subsequent treatment in case of relapse. In theory, tumor-specific T-cells induced by adjuvant DC vaccination might result in an increased tumor-specific immune response when ICI are given at a later moment in the metastatic setting. Indeed, this effect has been observed retrospectively with administration of ipilimumab in patients with relapse after adjuvant DC vaccination for stage III melanoma (60). In addition to ipilimumab, a similar effect was also seen retrospectively in glioblastoma (GBM) patients receiving chemotherapy after DC vaccination (61). These additive effects should be considered when integrating DC vaccines in the therapeutic landscape of cancer. Considering these arguments, the next part will focus on data obtained with DC vaccines in the adjuvant setting.

### Adjuvant DC vaccination in glioblastoma

Adjuvant DC vaccination has been studied in GBM. In contrast to most malignancies, distant metastases seldom occur in GBM (62). Nonetheless, GBM represents a lethal disease, with patients having a median survival of ~15 months (63). GBM is commonly treated with maximally safe surgery and adjuvant temozolamide (TMZ) in conjunction with radiotherapy, the so-called Stupp protocol (64). However, even with extensive treatment, residual disease invariably remains, and recurrence is certain. This results from the infiltrative growth and lack of a distinct border between normal brain tissue and tumor. Therefore, DC vaccination in the adjuvant setting after surgery in GBM is different from for example adjuvant DC vaccination in RCC and melanoma in which complete disease control after surgery is possible. In this review, we consider DC vaccination to be adjuvant when it is integrated in treatment protocols after maximally safe surgery in newly diagnosed GBM.

Historically, the central nervous system is considered an immune-privileged site, casting doubt whether GBM could be susceptible to immunotherapy. However, in recent years it has become increasingly clear the central nervous system is subject to active immunosurveillance even with an intact blood-brain barrier (65). Albeit not yet vigorously explored, the research into the treatment of GBM with ICI has not yet resulted in proof of efficacy. Nivolumab is the ICI furthest in clinical development, a phase III trial comparing nivolumab to bevacizumab for the first recurrence after radiotherapy and TMZ is currently ongoing (NCT02017717). Final results are not yet reported in a peer-reviewed journal, but presented results revealed that the primary end-point was not met (median OS in recurrent disease: 9.8 months with nivolumab vs. 10.0 months with bevacizumab) (66). Individual reports of response on anti-PD-1 mAb monotherapy do exist, although these are isolated cases concerning tumors with high mutational load (67–69). With these results in mind and the fact that mutational load and number of tumor infiltrating lymphocytes in GBM are generally low, it is doubtful whether ICI as monotherapy have promise as a future treatment option (70, 71).

Next to monotherapy with ICI, ICI combined with other standard treatment modalities is being investigated in phase III trials. For example, CheckMate 498 (comparing TMZ and radiotherapy to nivolumab and radiotherapy) and the CheckMate 548 (comparing radiotherapy, TMZ, and nivolumab to radiotherapy, TMZ and placebo), both involving nivolumab, are currently ongoing. Similar phase I and II trials combining pembrolizumab or ipilimumab with TMZ and radiotherapy are being performed. Results on such integration of ICI in standard treatment strategies are not yet reported.

Considering DC vaccination studies concerning GBM, DC-based therapy is often integrated into the standard adjuvant treatment for GBM. As of now, the only available phase III trial data involving DC vaccines in GBM are the very recently published interim results of an ongoing clinical study involving a vaccine called DCVax®-L (see also Table 2) (72). DCVax®-L is a vaccine manufactured from autologous DC loaded with tumor lysate derived from autologous GBM cells. Unblinded data on 331 patients with newly diagnosed GBM was presented. After surgery, patients were randomized (2:1) to receive either DCVax®-L incorporated into standard of care (TMZ and radiotherapy) or standard of care alone. Due to the study design, which enabled crossover from the standard of care to the vaccination arm upon progression, a total of 86% of patients received vaccination at the time of interim analysis. The authors compare the median OS of 23.1 months for the entire study population with OS data from comparable patients in different trials (which have a reported median OS of 15–17 months), from this comparison they suggest a clinical benefit from their vaccine. The definite results on clinical outcome, including PFS data, are eagerly awaited.

**Table 2:** Active phase III clinical trials concerning dendritic cell vaccination as adjuvant treatment in various malignancies (as of May 2018).

**Disease**	**Vaccine formulation**	**Status**	**Identifier**
Melanoma (stage III)	Natural dendritic cell subsets loaded with melanoma-specific peptides	Recruiting	NCT02993315
Uveal melanoma (high risk)	Dendritic cells loaded with autologous tumor RNA	Recruiting	NCT01983748
Glioblastoma (newly diagnosed)	DCVax®-L: dendritic cells loaded with tumor lysate	Active, not recruiting	NCT00045968

Previously, the favorable toxicity profile of DC vaccination was shown in several phase I/II studies showing the safety of adjuvant DC vaccination in GBM (73–78). Important to consider is that in these studies, DC vaccination was often combined with chemotherapy and/or radiotherapy, this combination had little added toxicity compared to chemotherapy and/or radiotherapy without DC vaccination. Despite not being designed for the purpose of assessing clinical outcome, these studies reported favorable median OS compared to their respective control groups ranging from 15 up to 41 months (74, 75, 77, 78). Furthermore, a positive correlation was shown between survival and presence of an immune response after vaccination (61).

Clinical outcome as primary endpoint was reported in several phase II studies. One of the largest studies completed to date involving DC vaccination in GBM, was performed by Ardon et al. and included 77 patients with newly diagnosed GBM (79). There was no control group, all patients received adjuvant DC vaccination integrated in standard treatment with TMZ and radiotherapy after complete resection of their GBM. The study reported favorable median OS of 18.3 months compared to the 14.6 months achieved in the landmark study by Stupp et al (64).

In conclusion, preliminary results on ICI in GBM make it very doubtful monotherapy with ICI will ever gain traction for this indication, results of large trials concerning ICI combined with chemoradiotherapy are pending. For DC vaccination in combination with chemoradiotherapy in GBM, occasionally favorable clinical outcomes have been reported. Due to strict inclusion criteria of these studies, the results are hard to interpret and compare with existing literature. Therefore, these result warrant further research with randomized phase III trials and additional data from the DCVax®-L trial are awaited.

### Adjuvant DC vaccination in RCC and melanoma

Besides GBM, both RCC and melanoma in certain stages also exhibit high recurrence rates after surgery. For melanoma, the risk of relapse is particularly high when the disease has metastasized to regional lymph nodes (stage III). Melanoma with lymph node metastasis has a 5-year survival rate ranging from 40% (stage IIIC) to 78% (stage IIIA) (80). In RCC, recurrence of disease following surgery is also common, resulting in a declining survival rate with increasing stage (81).

Melanoma and RCC are similar in the sense that both tumors are very chemo-resistant and that their adjuvant treatment strategy in the pre-ICI era was mainly based on cytokine treatment with IL-2 and IFN-α (82, 83). In both cancers, IL-2 and IFN-α provide little clinical benefit and are associated with high toxicity. For melanoma, ipilimumab showed clinical activity in the adjuvant setting with a 5-year recurrence-free survival rate of 41% compared to 30% in the placebo group (hazard ratio for recurrence or death, 0.76; p < 0.001). Importantly, 5-year distant metastasis-free survival rate was also improved with 48% compared to 39% (hazard ratio for death or distant metastasis, 0.76; *p* = 0.002) (21). Although these results show efficacy, the application of adjuvant ipilimumab is opposed by its significant toxicity (~40% of patients experience immune-related grade 3 or 4 adverse events) (21, 84). In addition, both nivolumab and pembrolizumab have shown to increase PFS in the adjuvant setting for melanoma (19, 20). Adjuvant nivolumab was tested against ipilimumab in completely resected stage IIIB, IIIC and IV melanoma. In this study adjuvant nivolumab improved the 1-year PFS rate to 72.3% compared to 61.6% in ipilimumab-treated patients. Similarly, adjuvant pembrolizumab was compared to placebo in stage IIIA, IIIB and IIIC melanoma. The 1-year PFS rates were 75% and 61%, respectively. Despite pending OS data, both the FDA and EMA recently granted approval for adjuvant nivolumab and are considering approval for adjuvant pembrolizumab.

For RCC, adjuvant treatment is also available. Adjuvant sunitinib, a tyrosine kinase inhibitor, for RCC has gained approval by the FDA based on improved PFS (6.8 months vs. 5.6 months for placebo; hazard ratio for recurrence, 0.76; *p* = 0.03). However, utility is limited due to high toxicity and lack of OS gain (85). Based on these considerations, the EMA has, in contrast to the FDA, adopted a negative opinion for the adjuvant application of sunitinib. In contrast to melanoma, for RCC no results on adjuvant ICI have been reported. However, several adjuvant clinical trials are ongoing, including the combination of ipilimumab and nivolumab (NCT03138512); atezolizumab (NCT03024996); pembrolizumab (NCT03142334) and nivolumab (NCT03055013) (82).

In both melanoma and RCC, DC vaccination has also been investigated as adjuvant treatment. Retrospective analysis from our group showed clinical benefit in stage III melanoma patients adjuvantly treated with monocyte-based DC vaccination compared to matched controls. In this study, OS for 78 patients treated with DC vaccines doubled compared to the 209 controls (63.6 months vs. 31.0 months; hazard ratio 0.59; p = 0.018) (58). Markowicz et al. have shown similar results in a prospective study concerning a peptide-loaded DC vaccine. In 22 vaccinated patients the study achieved a 3-year OS of 68% compared to 26% in the 22 patients of the matched historical control group (*p* = 0.029). The primary endpoint however, 3-year PFS rate, was not significantly improved probably due to the small number of patients (vaccinated patients: 41%; controls 15%; *p* = 0.108) (86). No phase III trials currently have been completed on adjuvant DC for melanoma. However, our group is currently conducting a trial which involves the employment of natural circulating DC vaccines in patients with stage IIIB or stage IIIC melanoma (NCT02993315) (Table 2).

In RCC, research on DC vaccination is mainly focused on metastatic disease and little data regarding adjuvant DC vaccination is available. However, a phase III trial was performed with adjuvant DC vaccination in various stages of disease. Patients vaccinated with DC loaded with tumor lysate in combination with cytokine-induced killer cells were compared to patients treated with IFN-α. Mainly due to a very heterogeneous study population, no definitive conclusions could be drawn. However, the study showed significant PFS and OS benefit suggesting that further research on adjuvant DC vaccination in RCC is warranted (87).

Currently, too little data is available to claim that DC vaccination is effective in the adjuvant setting. Yet, the above presented data, show favorable clinical results and consistently confirm the limited toxicity in a variety of cancers. More robust prove of efficacy may be under way as several phase III trials on adjuvant DC vaccination are currently being performed (Table 2). Whether DC vaccination acquires a definitive role in the adjuvant treatment of cancer will also be dependent on the results of ongoing phase III trials assessing other adjuvant treatments, including trials with ICI (88).

### The combination of DC vaccination and other modalities for the treatment of metastatic disease

**Figure 4:**
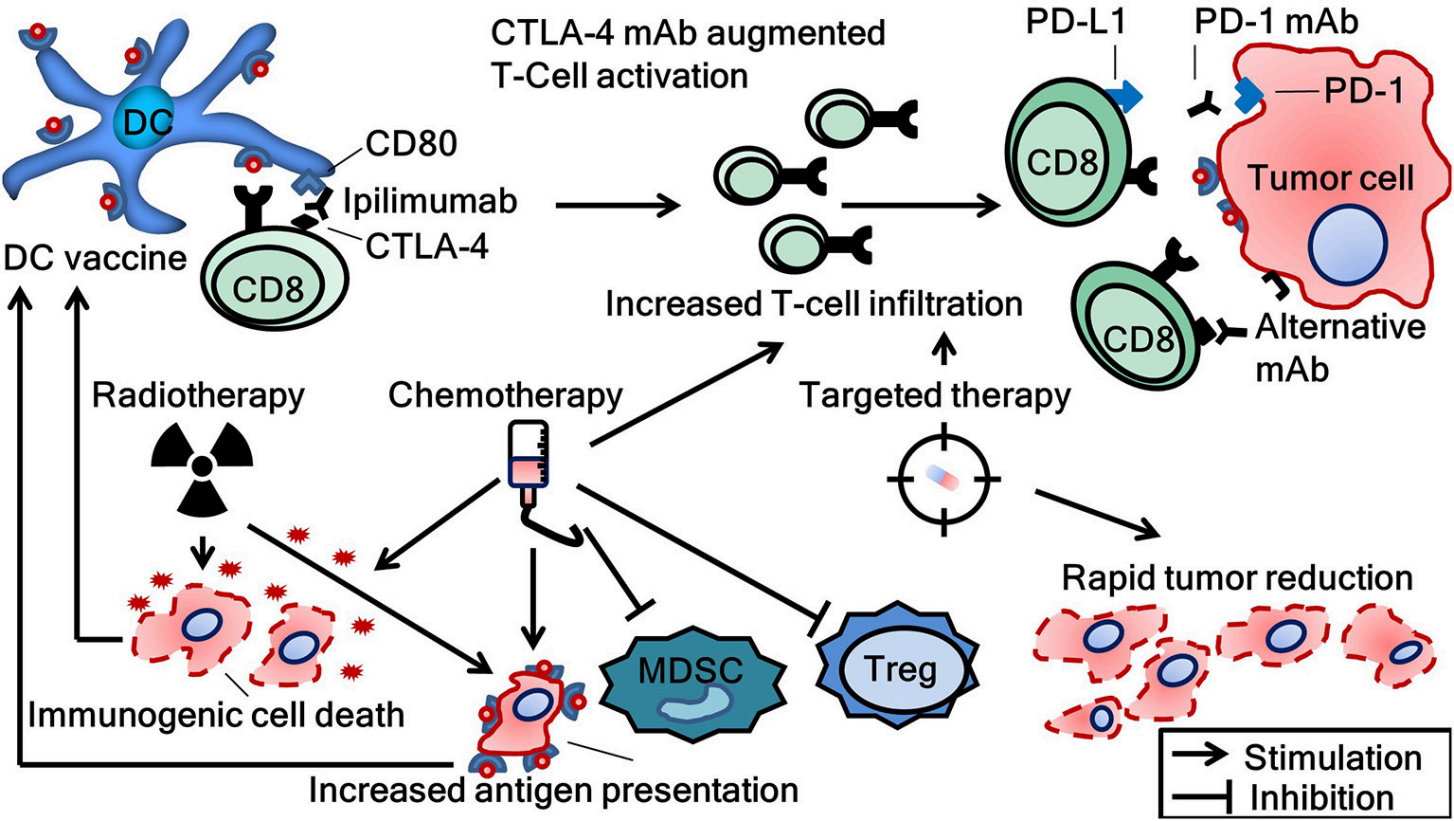
Combinational strategies to achieve synergy between several treatment modalities and dendritic cell vaccination. CTLA-4, cytotoxic T-lymphocyte-associated protein 4; DC, dendritic cell; mAb, monoclonal antibody; CD8, cluster of differentiation number 8 (cytotoxic T-cell); MDSC, myeloid derived suppressor cells; PD-1, programmed cell death protein; PD-L1, programmed death-ligand 1; Treg, regulatory T-cells.

As noted before, the clinical benefit of monotherapy DC vaccination for patients with metastatic disease is probably limited. However, the ultimate role for vaccines may lie in the combination with other modalities. The generation of a cellular immune response upon DC vaccination is commonly reported and may potentiate the effect of other anti-cancer therapeutics (23). Conversely, tumor reduction caused by chemotherapy, radiation therapy or targeted therapy can alleviate tumor-induced immune suppression which hinders efficacy of DC vaccination. However, possible synergies involve more than the mere reduction of tumor load as modalities other than immunotherapy also exhibit immunogenic effects on tumors (Figure 4). For example, although chemotherapeutics are associated with lymphodepletion, positive immune modulatory effects are described, including the induction of immunogenic cell death and depletion of Treg and MDSC (89–92). In addition, radiotherapy and different forms of targeted therapy are known to have immunostimulatory properties, i.e., enhanced T-cell infiltration and killing capacity (93–96). Clinical studies combining DC vaccination with chemotherapy, radiotherapy, and/or targeted therapy have been performed. Without extensive elaboration on these studies, the safety of combining DC vaccination with these modalities is confirmed in phase I trials (97–100). Futhermore, ample data exist suggesting efficacy (101, 102). Besides these treatment modalities, the combination of DC vaccination with other forms of immunotherapy intervening in additional steps of the cancer immunity cycle may be of particular interest as it is thought to result in more additive immunogenic effects. For example, it would be very interesting to explore the combination of DC vaccination with chimeric antigen receptor (CAR) T-cell therapy, oncolytic viruses, or other investigational immunotherapies. Here, we will discuss the combination of DC vaccination with the most successful immunotherapeutic agents to date, ICI.

Both ICI and DC vaccination exert their effects primarily through the modulation of the immune system and do so on different steps in the cancer immunity cycle. For response on ICI, tumor-specific T-cells have to be present in the tumor microenvironment, the generation of which may be aided with DC vaccination (103). As introduced before, a higher number of tumor-infiltrating lymphocytes are associated with a better response on ICI. In this respect, especially in tumors with low mutational burden, the addition of DC vaccines could prove to be beneficial (104).

Conversely, T-cells induced by DC vaccination are often hindered by the immune suppressive milieu of tumors. ICI might aid the effector functions of these T-cells by reducing inhibition through PD-1 signaling or by enhancing T-cell activation through the modulation of CTLA-4. The idea that tumor-specific T-cells activated by DC vaccination can be further stimulated with ICI is also supported by pre-clinical data. For example, upregulation of PD-1 on T-cells derived from the blood of vaccinated patients has been shown *in vitro* (105). Subsequent blockade of these upregulated PD-1 molecules could augment T-cell function. In addition, ICI exert several immune augmenting effects besides the direct antagonism of PD-1 and CTLA-4. For example, Treg depletion by anti-PD-1 mAb was shown in a mouse model (106).

In contrast to preclinical data, clinical data on combined treatment with ICI and DC vaccination in humans is scarce. In 2009, Ribas et al. reported safety of combining tremelimumab (CTLA-4 mAb) and DC vaccination in melanoma patients (107). Despite the trial was not designed to assess clinical outcome, 4 out of 16 patients (25%) achieved an objective clinical response. The authors state that clinical benefit was at the higher end of what can be expected from monotherapy tremelimumab. In addition, Wilgenhof et al. showed a promising ORR of 38% in 39 metastatic melanoma patients treated with the combination of ipilimumab and DC vaccination (108). In 36% of patients grade 3 or 4 adverse events were seen, which is comparable with rates seen in large clinical trials with monotherapy ipilimumab (5, 84). This suggests little added toxicity from the addition of DC vaccines to ICI.

Considering its lower toxicity and better response rates compared to anti-CTLA-4 mAb, anti-PD-1 mAb might be more suitable combinational partners for DC vaccines. As of now, no data is published on the combined anti-PD-1 mAb and DC vaccination. However, several clinical trials investigating combinations of DC vaccination with clinically approved ICI are currently being performed (Table 3).

**Table 3:** Ongoing clinical trials concerning dendritic cell vaccination in combination with clinically approved immune checkpoint inhibitors (ipilimumab, nivolumab, pembrolizumab, avelumab, atezolimumab, and durvalumab) in solid tumors.

**Immune checkpoint inhibition (target molecule)**	**Vaccine formulation**	**Malignancy**	**Status**	**NCT-identifier**
Combined Ipilimumab and Nivolumab (CTLA-4/PD-1)	DC with the insertion of the p53 gene	SCLC	Recruiting	NCT03406715
Nivolumab (PD-1)	DC loaded with CMV pp65 mRNA	Recurrent brain tumors	Active, not recruiting	NCT02529072
Nivolumab (PD-1)	DC loaded with NY-ESO-1 peptide	NY-ESO-1^+^ solid tumors	Recruiting	NCT02775292
Nivolumab (PD-1)	DC loaded with autologous tumor lysate	Recurrent glioblastoma	Not yet recruiting	NCT03014804
Pembrolizumab (PD-1)	DC loaded with peptide	Melanoma	Recruiting	NCT03092453
Pembrolizumab (PD-1)	DC-CIK	Solid tumors, NSCLC, Mesothelioma	Recruiting	NCT03190811 NCT03360630 NCT03393858
Nivolumab or Pembrolizumab (PD-1)	DC-CIK	Refractory solid tumors	Recruiting	NCT02886897
Avelumab (PD-L1)	DC/AML fusion vaccine	Colorectal cancer	Not yet recruiting	NCT03152565

*CIK, cytokine induced natural killer cells; CMV, cytomegalovirus; CTLA-4, cytotoxic T-lymphocyte-associated protein 4; DC, dendritic cells; NSCLC, non-small-cell lung cancer; SCLC, small-cell lung cancer; PD-1, programmed death-1; PD-L1, programmed death ligand 1*.

Besides currently approved ICI, DC vaccination can also be combined with ICI targeting alternative immune checkpoints (not -yet- clinically approved mAb). Currently, mAb targeting LAG-3 and TIM-3 are in various stages of clinical development as monotherapy and might be good candidates for combination. LAG-3 mAb for example, were shown to reduce expansion of Treg (109). TIM-3 was shown to be present in conjunction with PD-1 on dysfunctional T-cells after vaccination, suggesting they might form a target for mAb in addition to anti-PD-1 (110). Finally, the combination of multiple ICI and DC vaccination might be a promising strategy, albeit requiring careful considerations concerning the related toxicities (111).

Despite several ongoing clinical trials, an important aspect of combinational strategies, the timing of administration, might be under-investigated. In theory, it would seem logical to first administer DC vaccines to generate tumor-specific T-cells and consequently release immune suppression with anti-PD-1 mAb. Conversely, the timing of administering DC vaccines and ipilimumab may be more complex as both ipilimumab and these vaccines exert their functions in the priming phase of T-cells. Indeed, in a pre-clinical prostate cancer model optimal response on ipilimumab was shown when given on the same day as vaccination (112). Whether the timing of anti-PD-1 mAb and DC vaccination is equally important is not known and forms an interesting subject for further research.

In conclusion, combinational strategies for the treatment of cancer incorporating DC vaccination are a promising field of research. Considering the favorable results on the combination of DC vaccination and anti-CTLA-4 mAb, the results on the currently ongoing combinational clinical trials with anti-PD-1 and anti-PD-L1 mAb are eagerly awaited.

## Conclusion

Immunotherapy for the treatment of cancer is a fast-moving field. It is important to determine the relative position of DC vaccination to other treatments in this rapidly evolving landscape. Ideally, patients can be selected based on biomarkers predictive for response to therapy. Currently, no predictive biomarkers for DC vaccine response are applied in the clinic to guide treatment decisions but the immune landscape of the tumor might hold promise. Also, few clinically useful predictive biomarkers for ICI are known. With the success of ICI and the lesser clinical benefit of DC vaccination in metastatic disease, it becomes increasingly clear that the future of DC vaccination in extensive metastatic disease as standalone treatment is probably limited. However, the immune-inducing properties of DC vaccination makes it a prime candidate for combination with other anti-cancer modalities, especially ICI. The currently ongoing research on DC vaccination combined with ICI such as anti-PD-1 mAb has to determine whether this combination has a future perspective. The theoretical basis and the promising clinical data on anti-CTLA-4 mAb combined with DC vaccination does imply this perspective exists. With its highly favorable toxicity profile, another application of DC vaccination might lie in the adjuvant setting. Furthermore, DC vaccination as monotherapy may be more effective in adjuvant setting compared to its application in metastatic setting.

Consequently, for DC vaccination to gain a definitive role in the therapeutic landscape of cancer, research should be focused on well-designed trials in the adjuvant setting, combinational strategies, and patient selection.

## Disclosure

WG received speaker's fees from Bayer and Bristol-Myers Squibb; WG participated in advisory boards of Amgen, Astellas, Bayer, Bristol-Myers, Dendreon, Squibb, and Sanofi. WG participated in *ad hoc* consultancy for Aglaia Biomedical Ventures; WG received research grants from Bayer; Astellas and Janssen-Cilag.

## Author contributions

WvW, KB, and IdV conception and design; WvW, KB, MB, GS, IdV, and WG writing, review, and/or revision of the manuscript.

### Conflict of interest statement

The authors declare that the research was conducted in the absence of any commercial or financial relationships that could be construed as a potential conflict of interest.

## References

[1] Kienle GS. Fever in cancer treatment: coley's therapy and epidemiologic observations. Glob Adv Health Med. (2012) 1:92–100. 10.7453/gahmj.2012.1.1.016 24278806 PMC3833486

[2] Kottschade LA. Incidence and management of immune-related adverse events in patients undergoing treatment with immune checkpoint inhibitors. Curr Oncol Rep. (2018) 20:24. 10.1007/s11912-018-0671-4 29511902

[3] Hodi FS O'Day SJ McDermott DF Weber RW Sosman JA Haanen JB. Improved survival with ipilimumab in patients with metastatic melanoma. N Engl J Med. (2010) 363:711–23. 10.1056/NEJMoa1003466 20525992 PMC3549297

[4] Robert C Long GV Brady B Dutriaux C Maio M Mortier L. Nivolumab in previously untreated melanoma without BRAF mutation. N Engl J Med. (2015) 372:320–30. 10.1056/NEJMoa1412082 25399552

[5] Rozeman EA Dekker TJA Haanen J Blank CU. Advanced melanoma: current treatment options, biomarkers, and future perspectives. Am J Clin Dermatol. (2017) 19:303–17. 10.1007/s40257-017-0325-6 29164492

[6] Borghaei H Paz-Ares L Horn L Spigel DR Steins M Ready NE. Nivolumab versus docetaxel in advanced nonsquamous non-small-cell lung cancer. N Engl J Med. (2015) 373:1627–39. 10.1056/NEJMoa1507643 26412456 PMC5705936

[7] Weber JS Hodi FS Wolchok JD Topalian SL Schadendorf D Larkin J. Safety profile of nivolumab monotherapy: a pooled analysis of patients with advanced melanoma. J Clin Oncol. (2017) 35:785–92. 10.1200/JCO.2015.66.1389 28068177

[8] Blank C Brown I Peterson AC Spiotto M Iwai Y Honjo T. PD-L1/B7H-1 inhibits the effector phase of tumor rejection by T cell receptor (TCR) transgenic CD8+ T cells. Cancer Res. (2004) 64:1140–5 10.1158/0008-5472.CAN-03-3259 14871849

[9] Robert L Tsoi J Wang X Emerson R Homet B Chodon T. CTLA4 blockade broadens the peripheral T-cell receptor repertoire. Clin Cancer Res. (2014) 20:2424–32. 10.1158/1078-0432.CCR-13-2648 24583799 PMC4008652

[10] Larkin J Chiarion-Sileni V Gonzalez R Grob JJ Cowey CL Lao CD Combined nivolumab and ipilimumab or monotherapy in untreated melanoma. N Engl J Med. (2015) 373:23–34. 10.1056/NEJMoa1504030 26027431 PMC5698905

[11] Robert C Schachter J Long GV Arance A Grob JJ Mortier L. Pembrolizumab versus ipilimumab in advanced melanoma. N Engl J Med. (2015) 372:2521–32. 10.1056/NEJMoa1503093 25891173

[12] Schachter J Ribas A Long GV Arance A Grob JJ Mortier L. Pembrolizumab versus ipilimumab for advanced melanoma: final overall survival results of a multicentre, randomised, open-label phase 3 study (KEYNOTE-006). Lancet (2017) 390:1853–62. 10.1016/S0140-6736(17)31601-X 28822576

[13] Larkin J Hodi FS Wolchok JD Combined nivolumab and ipilimumab or monotherapy in untreated melanoma. N Engl J Med. (2015) 373:1270–1. 10.1056/NEJMc1509660 26398076

[14] Rittmeyer A Barlesi F Waterkamp D Park K Ciardiello F von Pawel J. Atezolizumab versus docetaxel in patients with previously treated non-small-cell lung cancer (OAK): a phase 3, open-label, multicentre randomised controlled trial. Lancet (2017) 389:255–65. 10.1016/S0140-6736(16)32517-X 27979383 PMC6886121

[15] Kim ES. Avelumab: first global approval. Drugs (2017) 77:929–37. 10.1007/s40265-017-0749-6 28456944

[16] Antonia SJ Villegas A Daniel D Vicente D Murakami S Hui R. Durvalumab after chemoradiotherapy in stage III non-small-cell lung cancer. N Engl J Med. (2017) 377:1919–29. 10.1056/NEJMoa1709937 28885881

[17] Vanpouille-Box C Lhuillier C Bezu L Aranda F Yamazaki T Kepp O. Trial watch: immune checkpoint blockers for cancer therapy. Oncoimmunology (2017) 6:e1373237. 10.1080/2162402X.2017.1373237 29147629 PMC5674958

[18] Torphy RJ Schulick RD Zhu Y. Newly emerging immune checkpoints: promises for future cancer therapy. Int J Mol Sci. (2017) 18:E2642. 10.3390/ijms18122642 29211042 PMC5751245

[19] Weber J Mandala M Del Vecchio M Gogas HJ Arance AM Cowey CL Adjuvant nivolumab versus ipilimumab in resected stage III or IV melanoma. N Engl J Med. (2017) 377:1824–35. 10.1056/NEJMoa1709030 28891423

[20] Eggermont AMM Blank CU Mandala M Long GV Atkinson V Dalle S. Adjuvant pembrolizumab versus placebo in resected stage III melanoma. N Engl J Med. (2018). 378:1789–801. 10.1056/NEJMoa1802357 29658430

[21] Eggermont AM Chiarion-Sileni V Grob JJ Dummer R Wolchok JD Schmidt H. Prolonged survival in stage III melanoma with ipilimumab adjuvant therapy. N Engl J Med. (2016) 375:1845–55. 10.1056/NEJMoa1611299 27717298 PMC5648545

[22] Weber JS Yang JC Atkins MB Disis ML. Toxicities of immunotherapy for the practitioner. J Clin Oncol. (2015) 33:2092–9. 10.1200/JCO.2014.60.0379 25918278 PMC4881375

[23] Draube A Klein-Gonzalez N Mattheus S Brillant C Hellmich M Engert A. Dendritic cell based tumor vaccination in prostate and renal cell cancer: a systematic review and meta-analysis. PLoS ONE (2011) 6:e18801. 10.1371/journal.pone.0018801 21533099 PMC3080391

[24] Anguille S Smits EL Lion E van Tendeloo VF Berneman ZN. Clinical use of dendritic cells for cancer therapy. Lancet Oncol. (2014) 15:e257–67. 10.1016/S1470-2045(13)70585-0 24872109

[25] de Vries IJ Bernsen MR Lesterhuis WJ Scharenborg NM Strijk SP Gerritsen MJ Immunomonitoring tumor-specific T cells in delayed-type hypersensitivity skin biopsies after dendritic cell vaccination correlates with clinical outcome. J Clin Oncol. (2005) 23:5779–87. 10.1200/JCO.2005.06.478 16110035

[26] Steinman RM Cohn ZA. Identification of a novel cell type in peripheral lymphoid organs of mice. I. Morphology, quantitation, tissue distribution. J Exp Med. (1973) 137:1142–62 4573839 10.1084/jem.137.5.1142 PMC2139237

[27] Banchereau J Steinman RM. Dendritic cells and the control of immunity. Nature (1998) 392:245–52. 10.1038/32588 9521319

[28] Wirth TC Harty JT Badovinac VP. Modulating numbers and phenotype of CD8+ T cells in secondary immune responses. Eur J Immunol. (2010) 40:1916–26. 10.1002/eji.201040310 20411564 PMC2993099

[29] Dhodapkar MV Steinman RM Krasovsky J Munz C Bhardwaj N. Antigen-specific inhibition of effector T cell function in humans after injection of immature dendritic cells. J Exp Med. (2001) 193:233–8 10.1084/jem.193.2.233 11208863 PMC2193335

[30] Sabado RL Balan S Bhardwaj N. Dendritic cell-based immunotherapy. Cell Res. (2017) 27:74–95. 10.1038/cr.2016.157 28025976 PMC5223236

[31] Bol KF Aarntzen EH Pots JM Olde Nordkamp MA van de Rakt MW Scharenborg NM. Prophylactic vaccines are potent activators of monocyte-derived dendritic cells and drive effective anti-tumor responses in melanoma patients at the cost of toxicity. Cancer Immunol Immunother. (2016) 65:327–39. 10.1007/s00262-016-1796-7 26861670 PMC4779136

[32] Bonehill A Van Nuffel AM Corthals J Tuyaerts S Heirman C Francois V. Single-step antigen loading and activation of dendritic cells by mRNA electroporation for the purpose of therapeutic vaccination in melanoma patients. Clin Cancer Res. (2009) 15:3366–75. 10.1158/1078-0432.CCR-08-2982 19417017

[33] Ott PA Hu Z Keskin DB Shukla SA Sun J Bozym DJ. An immunogenic personal neoantigen vaccine for patients with melanoma. Nature (2017) 547:217–21. 10.1038/nature22991 28678778 PMC5577644

[34] Tel J Anguille S Waterborg CE Smits EL Figdor CG de Vries IJ. Tumoricidal activity of human dendritic cells. Trends Immunol. (2014) 35:38–46. 10.1016/j.it.2013.10.007 24262387 PMC7106406

[35] Schuler-Thurner B Schultz ES Berger TG Weinlich G Ebner S Woerl P. Rapid induction of tumor-specific type 1 T helper cells in metastatic melanoma patients by vaccination with mature, cryopreserved, peptide-loaded monocyte-derived dendritic cells. J Exp Med. (2002) 195:1279–88 10.1084/jem.20012100 12021308 PMC2193752

[36] Banchereau J Palucka AK Dhodapkar M Burkeholder S Taquet N Rolland A. Immune and clinical responses in patients with metastatic melanoma to CD34(+) progenitor-derived dendritic cell vaccine. Cancer Res. (2001) 61:6451–8. 11522640

[37] Gibney GT Weiner LM Atkins MB. Predictive biomarkers for checkpoint inhibitor-based immunotherapy. Lancet Oncol. (2016) 17:e542–51. 10.1016/S1470-2045(16)30406-5 27924752 PMC5702534

[38] Buder-Bakhaya K Hassel JC. Biomarkers for clinical benefit of immune checkpoint inhibitor treatment-a review from the melanoma perspective and beyond. Front Immunol. (2018) 9:1474. 10.3389/fimmu.2018.01474 30002656 PMC6031714

[39] Vasaturo A Halilovic A Bol KF Verweij DI Blokx WA Punt CJ. T-cell landscape in a primary melanoma predicts the survival of patients with metastatic disease after their treatment with dendritic cell vaccines. Cancer Res. (2016) 76:3496–506. 10.1158/0008-5472.CAN-15-3211 27197179

[40] Chen DS Mellman I. Oncology meets immunology: the cancer-immunity cycle. Immunity (2013) 39:1–10. 10.1016/j.immuni.2013.07.012 23890059

[41] Motzer RJ Escudier B McDermott DF George S Hammers HJ Srinivas S. Nivolumab versus everolimus in advanced renal-cell carcinoma. N Engl J Med. (2015) 373:1803–13. 10.1056/NEJMoa1510665 26406148 PMC5719487

[42] Beer TM Kwon ED Drake CG Fizazi K Logothetis C Gravis G Randomized, double-blind, phase III trial of ipilimumab versus placebo in asymptomatic or minimally symptomatic patients with metastatic chemotherapy-naive castration-resistant prostate cancer. J Clin Oncol. (2017) 35:40–7. 10.1200/JCO.2016.69.1584 28034081

[43] Kwon ED Drake CG Scher HI Fizazi K Bossi A van den Eertwegh AJ. Ipilimumab versus placebo after radiotherapy in patients with metastatic castration-resistant prostate cancer that had progressed after docetaxel chemotherapy (CA184-043): a multicentre, randomised, double-blind, phase 3 trial. Lancet Oncol. (2014) 15:700–12. 10.1016/S1470-2045(14)70189-5 24831977 PMC4418935

[44] Guedes LB Antonarakis ES Schweizer MT Mirkheshti N Almutairi F Park JC. MSH2 loss in primary prostate cancer. Clin Cancer Res. (2017) 23:6863–74. 10.1158/1078-0432.CCR-17-0955 28790115 PMC5690834

[45] Graff JN Alumkal JJ Drake CG Thomas GV Redmond WL Farhad M. Early evidence of anti-PD-1 activity in enzalutamide-resistant prostate cancer. Oncotarget (2016) 7:52810–7. 10.18632/oncotarget.10547 27429197 PMC5288150

[46] Bishop JL Sio A Angeles A Roberts ME Azad AA Chi KN. PD-L1 is highly expressed in enzalutamide resistant prostate cancer. Oncotarget (2015) 6:234–42. 10.18632/oncotarget.2703 25428917 PMC4381591

[47] Boudadi K Suzman DL Luber B Wang H Silberstein J Sullivan R Phase 2 biomarker-driven study of ipilimumab plus nivolumab (Ipi/Nivo) for ARV7-positive metastatic castrate-resistant prostate cancer (mCRPC). J Clin Oncol. (2017) 35(Suppl. 15):5035. 10.1200/JCO.2017.35.15_suppl.5035

[48] Kantoff PW Higano CS Shore ND Berger ER Small EJ Penson DF. Sipuleucel-T immunotherapy for castration-resistant prostate cancer. N Engl J Med. (2010) 363:411–22. 10.1056/NEJMoa1001294 20818862

[49] Small EJ Schellhammer PF Higano CS Redfern CH Nemunaitis JJ Valone FH. Placebo-controlled phase III trial of immunologic therapy with sipuleucel-T (APC8015) in patients with metastatic, asymptomatic hormone refractory prostate cancer. J Clin Oncol. (2006) 24:3089–94. 10.1200/JCO.2005.04.5252 16809734

[50] Sheikh NA Petrylak D Kantoff PW Dela Rosa C Stewart FP Kuan LY. Sipuleucel-T immune parameters correlate with survival: an analysis of the randomized phase 3 clinical trials in men with castration-resistant prostate cancer. Cancer Immunol Immunother. (2013) 62:137–47. 10.1007/s00262-012-1317-2 22865266 PMC3541926

[51] Pantel K Riethmuller G. Micrometastasis detection and treatment with monoclonal antibodies. Curr Top Microbiol Immunol. (1996) 213(Pt 3):1–18. 8814999 10.1007/978-3-642-80071-9_1

[52] Lindau D Gielen P Kroesen M Wesseling P Adema GJ. The immunosuppressive tumour network: myeloid-derived suppressor cells, regulatory T cells and natural killer T cells. Immunology (2013) 138:105–15. 10.1111/imm.12036 23216602 PMC3575763

[53] Sabat R Grutz G Warszawska K Kirsch S Witte E Wolk K. Biology of interleukin-10. Cytokine Growth Factor Rev. (2010) 21:331–44. 10.1016/j.cytogfr.2010.09.002 21115385

[54] Yang L. TGFbeta, a potent regulator of tumor microenvironment and host immune response, implication for therapy. Curr Mol Med. (2010) 10:374–80 10.2174/156652410791317039 20455854

[55] Johnson BF Clay TM Hobeika AC Lyerly HK Morse MA. Vascular endothelial growth factor and immunosuppression in cancer: current knowledge and potential for new therapy. Expert Opin Biol Ther. (2007) 7:449–60. 10.1517/14712598.7.4.449 17373897

[56] Hornyak L Dobos N Koncz G Karanyi Z Pall D Szabo Z. The role of indoleamine-2,3-dioxygenase in cancer development, diagnostics, and therapy. Front Immunol. (2018) 9:151. 10.3389/fimmu.2018.00151 29445380 PMC5797779

[57] Gulley JL Madan RA Schlom J. Impact of tumour volume on the potential efficacy of therapeutic vaccines. Curr Oncol. (2011) 18:e150–7. 10.3747/co.v18i3.783 21655153 PMC3108875

[58] Bol KF Aarntzen EH Hout FE Schreibelt G Creemers JH Lesterhuis WJ. Favorable overall survival in stage III melanoma patients after adjuvant dendritic cell vaccination. Oncoimmunology (2016) 5:e1057673. 10.1080/2162402X.2015.1057673 26942068 PMC4760342

[59] Aarntzen EH Bol K Schreibelt G Jacobs JF Lesterhuis WJ Van Rossum MM. Skin-test infiltrating lymphocytes early predict clinical outcome of dendritic cell-based vaccination in metastatic melanoma. Cancer Res. (2012) 72:6102–10. 10.1158/0008-5472.CAN-12-2479 23010076

[60] Boudewijns S Koornstra RH Westdorp H Schreibelt G van den Eertwegh AJ Geukes Foppen MH. Ipilimumab administered to metastatic melanoma patients who progressed after dendritic cell vaccination. Oncoimmunology (2016) 5:e1201625. 10.1080/2162402X.2016.1201625 27622070 PMC5007966

[61] Wheeler CJ Das A Liu G Yu JS Black KL. Clinical responsiveness of glioblastoma multiforme to chemotherapy after vaccination. Clin Cancer Res. (2004) 10:5316–26. 10.1158/1078-0432.CCR-04-0497 15328167

[62] Schweitzer T Vince GH Herbold C Roosen K Tonn JC. Extraneural metastases of primary brain tumors. J Neurooncol. (2001) 53:107–14 10.1023/A:1012245115209 11716064

[63] Stupp R Hegi ME Mason WP van den Bent MJ Taphoorn MJ Janzer RC. Effects of radiotherapy with concomitant and adjuvant temozolomide versus radiotherapy alone on survival in glioblastoma in a randomised phase III study: 5-year analysis of the EORTC-NCIC trial. Lancet Oncol. (2009) 10:459–66. 10.1016/S1470-2045(09)70025-7 19269895

[64] Stupp R Mason WP van den Bent MJ Weller M Fisher B Taphoorn MJ. Radiotherapy plus concomitant and adjuvant temozolomide for glioblastoma. N Engl J Med. (2005) 352:987–96. 10.1056/NEJMoa043330 15758009

[65] Lim M Xia Y Bettegowda C Weller M. Current state of immunotherapy for glioblastoma. Nat Rev Clin Oncol. (2018). 15:422–42. 10.1038/s41571-018-0003-5 29643471

[66] Reardon DA Omuro A Brandes AA Rieger J Wick A Sepulveda J OS10.3 Randomized phase 3 study evaluating the efficacy and safety of nivolumab vs bevacizumab in patients with recurrent glioblastoma: checkmate 143. Neuro-Oncology. (2017) 19(Suppl. 3):iii21. 10.1093/neuonc/nox036.071

[67] Roth P Valavanis A Weller M. Long-term control and partial remission after initial pseudoprogression of glioblastoma by anti-PD-1 treatment with nivolumab. Neuro Oncol. (2017) 19:454–6. 10.1093/neuonc/now265 28039369 PMC5464329

[68] Bouffet E Larouche V Campbell BB Merico D de Borja R Aronson M. Immune checkpoint inhibition for hypermutant glioblastoma multiforme resulting from germline biallelic mismatch repair deficiency. J Clin Oncol. (2016) 34:2206–11. 10.1200/JCO.2016.66.6552 27001570

[69] Johanns TM Miller CA Dorward IG Tsien C Chang E Perry A. Immunogenomics of hypermutated glioblastoma: a patient with germline POLE deficiency treated with checkpoint blockade immunotherapy. Cancer Discov. (2016) 6:1230–6. 10.1158/2159-8290.CD-16-0575 27683556 PMC5140283

[70] Hao C Parney IF Roa WH Turner J Petruk KC Ramsay DA. Cytokine and cytokine receptor mRNA expression in human glioblastomas: evidence of Th1, Th2 and Th3 cytokine dysregulation. Acta Neuropathol. (2002) 103:171–8. 10.1007/s004010100448 11810184

[71] Hodges TR Ott M Xiu J Gatalica Z Swensen J Zhou S. Mutational burden, immune checkpoint expression, and mismatch repair in glioma: implications for immune checkpoint immunotherapy. Neuro Oncol. (2017) 19:1047–57. 10.1093/neuonc/nox026 28371827 PMC5570198

[72] Liau LM Ashkan K Tran DD Campian JL Trusheim JE Cobbs CS First results on survival from a large Phase 3 clinical trial of an autologous dendritic cell vaccine in newly diagnosed glioblastoma. J Trans Med. (2018) 16:142 10.1186/s12967-018-1507-6 PMC5975654 29843811

[73] Olin MR Low W McKenna DH Haines SJ Dahlheimer T Nascene D. Vaccination with dendritic cells loaded with allogeneic brain tumor cells for recurrent malignant brain tumors induces a CD4(+)IL17(+) response. J Immunother Cancer (2014) 2:4. 10.1186/2051-1426-2-4 24829761 PMC4019901

[74] Yu JS Wheeler CJ Zeltzer PM Ying H Finger DN Lee PK. Vaccination of malignant glioma patients with peptide-pulsed dendritic cells elicits systemic cytotoxicity and intracranial T-cell infiltration. Cancer Res. (2001) 61:842–7. 11221866

[75] Phuphanich S Wheeler CJ Rudnick JD Mazer M Wang H Nuno MA. Phase I trial of a multi-epitope-pulsed dendritic cell vaccine for patients with newly diagnosed glioblastoma. Cancer Immunol Immunother. (2013) 62:125–35. 10.1007/s00262-012-1319-0 22847020 PMC3541928

[76] Kamigaki T Kaneko T Naitoh K Takahara M Kondo T Ibe H. Immunotherapy of autologous tumor lysate-loaded dendritic cell vaccines by a closed-flow electroporation system for solid tumors. Anticancer Res. (2013) 33:2971–6. 23780988

[77] Liau LM Prins RM Kiertscher SM Odesa SK Kremen TJ Giovannone AJ. Dendritic cell vaccination in glioblastoma patients induces systemic and intracranial T-cell responses modulated by the local central nervous system tumor microenvironment. Clin Cancer Res. (2005) 11:5515–25. 10.1158/1078-0432.CCR-05-0464 16061868

[78] Batich KA Reap EA Archer GE Sanchez-Perez L Nair SK Schmittling RJ. Long-term survival in glioblastoma with *Cytomegalovirus* pp65-targeted vaccination. Clin Cancer Res. (2017) 23:1898–909. 10.1158/1078-0432.CCR-16-2057 28411277 PMC5559300

[79] Ardon H Van Gool SW Verschuere T Maes W Fieuws S Sciot R. Integration of autologous dendritic cell-based immunotherapy in the standard of care treatment for patients with newly diagnosed glioblastoma: results of the HGG-2006 phase I/II trial. Cancer Immunol Immunother. (2012) 61:2033–44. 10.1007/s00262-012-1261-1 22527250 PMC11028710

[80] Balch CM Gershenwald JE Soong SJ Thompson JF Atkins MB Byrd DR. Final version of 2009 AJCC melanoma staging and classification. J Clin Oncol. (2009) 27:6199–206. 10.1200/JCO.2009.23.4799 19917835 PMC2793035

[81] Amin MB Edge SB Greene F Byrd DR Brookland RK Washington MK. AJCC Cancer Staging Manual. New York, NY: Springer International Publishing (2017). p. 479.

[82] Massari F Di Nunno V Ciccarese C Graham J Porta C Comito F. Adjuvant therapy in renal cell carcinoma. Cancer Treat Rev. (2017) 60:152–7. 10.1016/j.ctrv.2017.09.004 28992528

[83] Verma S Quirt I McCready D Bak K Charette M Iscoe N. Systematic review of systemic adjuvant therapy for patients at high risk for recurrent melanoma. Cancer (2006) 106:1431–42. 10.1002/cncr.21760 16511841

[84] Eggermont AM Chiarion-Sileni V Grob JJ Dummer R Wolchok JD Schmidt H Adjuvant ipilimumab versus placebo after complete resection of high-risk stage III melanoma (EORTC 18071): a randomised, double-blind, phase 3 trial. Lancet Oncol. (2015) 16:522–30. 10.1016/S1470-2045(15)70122-1 25840693

[85] Ravaud A Motzer RJ Pandha HS George DJ Pantuck AJ Patel A. Adjuvant sunitinib in high-risk renal-cell carcinoma after nephrectomy. N Engl J Med. (2016) 375:2246–54. 10.1056/NEJMoa1611406 27718781

[86] Markowicz S Nowecki ZI Rutkowski P Lipkowski AW Biernacka M Jakubowska-Mucka A. Adjuvant vaccination with melanoma antigen-pulsed dendritic cells in stage III melanoma patients. Med Oncol. (2012) 29:2966–77. 10.1007/s12032-012-0168-1 22302285

[87] Zheng K Tan JM Wu WZ Qiu YM Zhang H Xu TZ. Adjuvant dendritic cells vaccine combined with cytokine-induced-killer cell therapy after renal cell carcinoma surgery. J BUON (2015) 20:505–13. 26011343

[88] Huang J Liu F Liu Z Tang H Wu H Gong Q. Immune checkpoint in glioblastoma: promising and challenging. Front Pharmacol. (2017) 8:242. 10.3389/fphar.2017.00242 28536525 PMC5422441

[89] Obeid M Tesniere A Panaretakis T Tufi R Joza N van Endert P. Ecto-calreticulin in immunogenic chemotherapy. Immunol Rev. (2007) 220:22–34. 10.1111/j.1600-065X.2007.00567.x 17979837

[90] Ghiringhelli F Menard C Puig PE Ladoire S Roux S Martin F. Metronomic cyclophosphamide regimen selectively depletes CD4+CD25+ regulatory T cells and restores T and NK effector functions in end stage cancer patients. Cancer Immunol Immunother. (2007) 56:641–8. 10.1007/s00262-006-0225-8 16960692 PMC11030569

[91] Galluzzi L Buque A Kepp O Zitvogel L Kroemer G. Immunological effects of conventional chemotherapy and targeted anticancer agents. Cancer Cell (2015) 28:690–714. 10.1016/j.ccell.2015.10.012 26678337

[92] Tongu M Harashima N Monma H Inao T Yamada T Kawauchi H. Metronomic chemotherapy with low-dose cyclophosphamide plus gemcitabine can induce anti-tumor T cell immunity *in vivo*. Cancer Immunol Immunother. (2013) 62:383–91. 10.1007/s00262-012-1343-0 22926062 PMC11029128

[93] Chakraborty M Abrams SI Camphausen K Liu K Scott T Coleman CN. Irradiation of tumor cells up-regulates Fas and enhances CTL lytic activity and CTL adoptive immunotherapy. J Immunol. (2003) 170:6338–47 10.4049/jimmunol.170.12.6338 12794167

[94] Garnett CT Palena C Chakraborty M Tsang KY Schlom J Hodge JW. Sublethal irradiation of human tumor cells modulates phenotype resulting in enhanced killing by cytotoxic T lymphocytes. Cancer Res. (2004) 64:7985–94. 10.1158/0008-5472.CAN-04-1525 15520206

[95] Wilmott JS Long GV Howle JR Haydu LE Sharma RN Thompson JF. Selective BRAF inhibitors induce marked T-cell infiltration into human metastatic melanoma. Clin Cancer Res. (2012) 18:1386–94. 10.1158/1078-0432.CCR-11-2479 22156613

[96] Koya RC Mok S Otte N Blacketor KJ Comin-Anduix B Tumeh PC. BRAF inhibitor vemurafenib improves the antitumor activity of adoptive cell immunotherapy. Cancer Res. (2012) 72:3928–37. 10.1158/0008-5472.CAN-11-2837 22693252 PMC3422880

[97] Matsushita H Enomoto Y Kume H Nakagawa T Fukuhara H Suzuki M. A pilot study of autologous tumor lysate-loaded dendritic cell vaccination combined with sunitinib for metastatic renal cell carcinoma. J Immunother Cancer (2014) 2:30. 10.1186/s40425-014-0030-4 25694811 PMC4331924

[98] Zhang L Xu Y Shen J He F Zhang D Chen Z Feasibility study of DCs/CIKs combined with thoracic radiotherapy for patients with locally advanced or metastatic non-small-cell lung cancer. Radiat Oncol. (2016) 11:60. 10.1186/s13014-016-0635-5 PMC4839093 27097970

[99] Yanagisawa R Koizumi T Koya T Sano K Koido S Nagai K. WT1-pulsed dendritic cell vaccine combined with chemotherapy for resected pancreatic cancer in a phase I study. Anticancer Res. (2018) 38:2217–25. 10.21873/anticanres.12464 29599342

[100] Laurell A Lonnemark M Brekkan E Magnusson A Tolf A Wallgren AC. Intratumorally injected pro-inflammatory allogeneic dendritic cells as immune enhancers: a first-in-human study in unfavourable risk patients with metastatic renal cell carcinoma. J Immunother Cancer (2017) 5:52. 10.1186/s40425-017-0255-0 28642820 PMC5477104

[101] Figlin RA. Personalized immunotherapy (AGS-003) when combined with sunitinib for the treatment of metastatic renal cell carcinoma. Expert Opin Biol Ther. (2015) 15:1241–8. 10.1517/14712598.2015.1063610 26125651

[102] Wang C Pu J Yu H Liu Y Yan H He Z. a dendritic cell vaccine combined with radiotherapy activates the specific immune response in patients with esophageal cancer. J Immunother. (2017) 40:71–6. 10.1097/CJI.0000000000000155 28125513

[103] Fong L Carroll P Weinberg V Chan S Lewis J Corman J. Activated lymphocyte recruitment into the tumor microenvironment following preoperative sipuleucel-T for localized prostate cancer. J Natl Cancer Inst. (2014) 106:dju268. 10.1093/jnci/dju268 25255802 PMC4241888

[104] Garg AD Coulie PG Van den Eynde BJ Agostinis P. Integrating next-generation dendritic cell vaccines into the current cancer immunotherapy landscape. Trends Immunol. (2017) 38:577–93. 10.1016/j.it.2017.05.006 28610825

[105] Fourcade J Sun Z Pagliano O Chauvin JM Sander C Janjic B. PD-1 and Tim-3 regulate the expansion of tumor antigen-specific CD8(+) T cells induced by melanoma vaccines. Cancer Res. (2014) 74:1045–55. 10.1158/0008-5472.CAN-13-2908 24343228 PMC3952491

[106] Dyck L Wilk MM Raverdeau M Misiak A Boon L Mills KH. Anti-PD-1 inhibits Foxp3(+) Treg cell conversion and unleashes intratumoural effector T cells thereby enhancing the efficacy of a cancer vaccine in a mouse model. Cancer Immunol Immunother. (2016) 65:1491–8. 10.1007/s00262-016-1906-6 27680570 PMC11028992

[107] Ribas A Comin-Anduix B Chmielowski B Jalil J de la Rocha P McCannel TA. Dendritic cell vaccination combined with CTLA4 blockade in patients with metastatic melanoma. Clin Cancer Res. (2009) 15:6267–76. 10.1158/1078-0432.CCR-09-1254 19789309 PMC2765061

[108] Wilgenhof S Corthals J Van Nuffel AM Benteyn D Heirman C Bonehill A. Long-term clinical outcome of melanoma patients treated with messenger RNA-electroporated dendritic cell therapy following complete resection of metastases. Cancer Immunol Immunother. (2015) 64:381–8. 10.1007/s00262-014-1642-8 25548092 PMC11029539

[109] Romano E Michielin O Voelter V Laurent J Bichat H Stravodimou A. MART-1 peptide vaccination plus IMP321 (LAG-3Ig fusion protein) in patients receiving autologous PBMCs after lymphodepletion: results of a Phase I trial. J Transl Med. (2014) 12:97. 10.1186/1479-5876-12-97 24726012 PMC4021605

[110] Fourcade J Sun Z Benallaoua M Guillaume P Luescher IF Sander C. Upregulation of Tim-3 and PD-1 expression is associated with tumor antigen-specific CD8+ T cell dysfunction in melanoma patients. J Exp Med. (2010) 207:2175–86. 10.1084/jem.20100637 20819923 PMC2947081

[111] Vasaturo A Di Blasio S Peeters DG de Koning CC de Vries JM Figdor CG. Clinical implications of co-inhibitory molecule expression in the tumor microenvironment for DC vaccination: a game of stop and go. Front Immunol. (2013) 4:417. 10.3389/fimmu.2013.00417 24348481 PMC3847559

[112] Wada S Jackson CM Yoshimura K Yen HR Getnet D Harris TJ. Sequencing CTLA-4 blockade with cell-based immunotherapy for prostate cancer. J Transl Med. (2013) 11:89. 10.1186/1479-5876-11-89 23557194 PMC3666941

